# Prevalence and impact of workplace violence against healthcare workers in Bosnia and Herzegovina

**DOI:** 10.3325/cmj.2025.66.376

**Published:** 2025-10

**Authors:** Vedad Herenda, Elvedina Žiga, Dženana Hrlović

**Affiliations:** 1 Nephrology Clinic, Clinical Center, University of Sarajevo, Sarajevo, Bosnia and Herzegovina; 2 Medical School, Sarajevo University School of Science and Technology, Sarajevo, Bosnia and Herzegovina; 3 Institute for Public Health of Federation Bosnia and Herzegovina, Sarajevo, Bosnia and Herzegovina; 4 NGO Menssana, Sarajevo, Bosnia and Herzegovina

Workplace violence (WPV) refers to “incidents in which workers are abused, threatened, or assaulted in circumstances related to their work, including commuting, and which involve an implicit or explicit risk to their safety, well-being, or health” (1). Due to their close interactions with patients, healthcare workers (HCWs) are at a heightened risk of aggression, which accounts for 75% of reported WPV incidents in the workplace. The prevalence of WPV varies by region, partly due to differences in reporting practices. While WPV is a recognized global issue, data from Southeastern Europe, particularly Bosnia and Herzegovina (B&H), are scarce. The absence of research suggests that WPV may be underrecognized and underreported. We aimed to assess the prevalence and forms of WPV among HCWs in B&H. To our knowledge, this is the first comprehensive investigation of WPV that includes the entire territory of B&H. The instrument, Workplace Violence in the Health Sector – Country Case Study Research Instruments – Survey Questionnaire, was obtained from the World Health Organization website. It was translated and modified following cross-cultural adaptation guidelines. We contacted 12 professional organizations via email. Sadly, only one organization agreed to distribute the survey to its members. Additionally, the study was promoted on social media, and HCWs were encouraged to invite colleagues to participate via an anonymous online questionnaire. The investigation involved 228 participants, out of whom 241 fully completed the questionnaires.

A high prevalence of WPV was observed among HCWs – 93.4% reported any form of violence during their career and 70.6% in the past 12 months. The findings suggest that WPV is a significant issue within the country’s healthcare system, likely affecting its quality and professional performance. An earlier study conducted in Sarajevo reported similar results (2). The underrepresentation of nurses in our sample may have biased the overall prevalence estimates, as nurses are widely recognized as a high-risk group for WPV. Our results indicate a higher prevalence of lifetime verbal violence than previously reported. However, one-year prevalence of physical violence was lower than the global average. Verbal abuse remains the most common form of WPV worldwide, occurring 2 to 10 times more frequently than physical violence. Similarly, most participants in our investigation reported having experienced verbal attacks (Table 1). A systematic review estimated the global prevalence of WPV against HCWs in low- and middle-income countries to range from 60.8% to 82.2% (3), while our study reported an even higher prevalence. Similar WPV rates have been observed in Georgia, Ethiopia, and Bangladesh (4-6). In Latin America, WPV is a recognized issue, yet the verbal violence rates reported in our study were higher than those observed in that region (7). Compared with neighboring Southeastern European countries, WPV in B&H appears more prevalent than in Bulgaria and Serbia (8,9). However, our study found lower rates of physical violence than reported in these countries. The prevalence of WPV in B&H is similar to that in North Macedonia (10). Data from Croatia suggest an overall WPV prevalence of 80% (11). In comparison, reports from Serbia indicate WPV rates of 52.6% in primary care settings and 57.5% in psychiatric wards, with physical violence occurring in 33.6% of cases (12). In North Macedonia, WPV prevalence has been reported at 73.5% (10). However, data from Kosovo and Montenegro are lacking, with only anecdotal reports from news sources. Consistent with previous studies (14), our findings confirm that the most common perpetrators of WPV are patients’ relatives (Table 2). Similar patterns have been observed in other countries, though a study from Turkey reported that the most frequent aggressors were the patients themselves (13).

**Table 1:** Overall prevalence of violence according to type

Violence type	Total (n)	Overall prevalence (%)	Last 12 months (n)	Percent
Any form of violence	213	93.4	161	70.6
Physical violence	68	29.8	25	11.0
Verbal abuse	211	92.5	161	70.6

**Table 2:** Overall prevalence of violence and according to healthcare level and perpetrator

	Frequency (n)	Percent (%)
**Perpetrator type**		
relatives of the patient	135	59.5
patients	92	40.5
**Violence by healthcare level**		
primary	87	84.5
secondary	29	67.4
tertiary	41	67.2

As key contributors to WPV, respondents identified organizational factors, particularly staff shortages and long waiting times. Another study reported that structural and systemic deficiencies were the primary drivers of WPV (15). Our investigation found the highest WPV prevalence in primary care settings (Table 2), while other research identified tertiary care, including emergency departments, psychiatric wards, and intensive care units, as the most affected settings (10). This discrepancy may stem from differences in facility organization. Hospitals in B&H employ security personnel, whereas primary healthcare facilities – often located in remote areas – lack such protection. Additionally, HCWs in primary care settings expressed significant concern about future trends, with most believing that WPV will worsen. Similar concerns have been observed elsewhere; for instance, WPV incidents in the United Kingdom’s primary care settings doubled over five years (16). If this trend continues, HCWs may opt for positions in more secure tertiary care settings, potentially undermining primary care services and reducing healthcare accessibility.

Despite the high prevalence of WPV, many HCWs in our study expressed dissatisfaction with institutional responses. Frequently cited barriers to reporting included a lack of formal procedures, distrust in reporting systems, and fear of retaliation. Additionally, most participants who reported WPV incidents were dissatisfied with how their cases were handled, a common concern in other studies. Inadequate institutional responses may erode morale and discourage future reporting. B&H lacks a unified law protecting HCWs against WPV. Existing labor and public health laws are vague and often poorly enforced, while the absence of strict penalties further complicates efforts to address this issue.

Without specific legal measures that acknowledge the unique challenges of the healthcare work environment, preventing and addressing WPV against HCWs will remain a significant challenge. Legal reforms criminalizing violence against HCWs could send a strong message that WPV is unacceptable. Additionally, the visible presence of security personnel, along with clearly posted warnings about zero-tolerance policies and legal consequences, is likely one of the most effective short-term deterrents.

## References

[1] International Labour Organization. Publications on International Labour Standards. Available from: https://www.ilo.org/global/standards/information-resources-and-publications/publications/lang–en/index.htm*.* Accessed: March 5, 2025.

[2] Jatic Z Erkocevic H Trifunovic N Tatarevic E Keco A Sporisevic L Frequency and forms of workplace violence in primary health care. Med Arch 2019 73 6 10 10.5455/medarh.2019.73.6-10 31097851 PMC6445619

[3] Rai R El-Zaemey S Dorji N Rai BD Fritschi L Exposure to occupational hazards among health care workers in low- and middle-income countries: a scoping review. Int J Environ Res Public Health 2021 18 2603 10.3390/ijerph18052603 33807727 PMC7967386

[4] Kareli M Pitskhelauri N 0085 Cross-sectional study – the prevalence and effects of workplace violence against medical staff in three hospitals of Tbilisi, Georgia. Inj Prev 2021 27 A24 10.1136/injuryprev-2021-SAVIR.62

[5] Bekalu YE Wudu MA Prevalence of workplace violence and associated factors against nurses working in public hospitals in Northeastern Ethiopia, 2022. SAGE Open Nurs 2023 9 23779608231171776 10.1177/23779608231171776 37250765 PMC10210530

[6] Khan Rony MK, Das Sharmi P, Parvin MR, Kayesh I, Alamgir HM. Prevalence and risk factors of workplace violence against healthcare workers in Bangladesh and its consequences: A nationwide cross-sectional study. Informatics in Medicine Unlocked. Volume 41, 2023.

[7] Mautong H, Camacho-Leon G, Khalid A, Huespe I, Bhatt G, Banga A. Workplace violence against healthcare workers in Latin America: a subgroup analysis from 12 countries of the Global ViSHWaS Study. Available from: https://papers.ssrn.com/sol3/papers.cfm?abstract_id=4567893*.* Accessed: October 31, 2025.

[8] Di Martino V. Workplace violence in the health sector-country case studies Brazil, Bulgaria, Lebonan, Portagal, South Africa, Thailand plus an additional Australian study. ILO/ICN/WHO/PSI joint programme on workplace violence in the health sector, 2002.

[9] Marina B Fisekovic, Goran Z. Trajkovic, Vesna M. Bjegovic-Mikanovic, Zorica J. Terzic-Supic, Does workplace violence exist in primary health care? Evidence from Serbia Eur J Public Health 2015 25 693 8 10.1093/eurpub/cku247 25644138

[10] Pekez-Pavlisko T Racic M Gavran L Pavlic DR Sukriev L Zivanovic SR Workplace violence and sanctioning of family medicine physicians due to the rules of health insurance funds in the Western Balkan. Mater Sociomed 2019 31 99 104 10.5455/msm.2019.31.99-104 31452633 PMC6690306

[11] de Raeve P Xyrichis A Bolzonella F Bergs J Davidson PM Workplace violence against nurses: challenges and solutions for Europe. Policy Polit Nurs Pract 2023 24 255 64 10.1177/15271544231182586 37475497 PMC10563370

[12] Terzic-Supic ZJ Fisekovic-Kremic MB Todorovic J Santric-Milicevic MM Nesic DM Trajkovic GZ Violence against primary health care workers in Serbia and measures for ensuring safe work environment. Iran J Public Health 2019 48 2304 5 31993403 PMC6974857

[13] Ipek M Özlük B Patients’ opinion on violence against healthcare workers and their level of satisfaction in emergency department in Turkey: A cross-sectional study. Int Emerg Nurs 2023 71 101350 10.1016/j.ienj.2023.101350 37708667

[14] Lim MC Jeffree MS Saupin SS Giloi N Lukman KA Workplace violence in healthcare settings: The risk factors, implications and collaborative preventive measures. Ann Med Surg (Lond) 2022 78 103727 10.1016/j.amsu.2022.103727 35734684 PMC9206999

[15] O’Brien CJ van Zundert AAJ Barach PR The growing burden of workplace violence against healthcare workers: trends in prevalence, risk factors, consequences, and prevention – a narrative review. EClinicalMedicine 2024 72 102641 10.1016/j.eclinm.2024.102641 38840669 PMC11152903

[16] Iacobucci G Violent incidents at GP surgeries double in five years, BMJ investigation finds. BMJ 2022 377 o1333 10.1136/bmj.o1333 35640962

